# Towards Equity in Health: Researchers Take Stock

**DOI:** 10.1371/journal.pmed.1002186

**Published:** 2016-11-29

**Authors:** The PLOS Medicine Editors, Annette Rid, Michael A. Johansson, Gabriel Leung, Hannah Valantine, Esteban G. Burchard, Sam S. Oh, Cathy Zimmerman

**Affiliations:** 1 Public Library of Science, San Francisco, California, United States of America; 2 Public Library of Science, Cambridge, United Kingdom; 3 Department of Global Health & Social Medicine, King’s College London, London, United Kingdom; 4 Division of Vector-Borne Diseases, Centers for Disease Control and Prevention, San Juan, Puerto Rico; 5 Center for Communicable Disease Dynamics, T.H. Chan School of Public Health, Boston, Massachusetts, United States of America; 6 Li Ka Shing Faculty of Medicine, The University of Hong Kong, Hong Kong, China; 7 Office of the Director, National Institutes of Health, Bethesda, Maryland, United States of America; 8 Laboratory of Genome Transplantation, National Heart, Lung, and Blood Institute, National Institutes of Health, Bethesda, Maryland, United States of America; 9 Department of Bioengineering & Therapeutic Sciences, University of California San Francisco, San Francisco, California, United States of America; 10 Department of Medicine, University of California San Francisco, San Francisco, California, United States of America; 11 Department of Global Health and Development, London School of Hygiene and Tropical Medicine, London, United Kingdom

## Abstract

For the 2016 end-of-the-year editorial, the *PLOS Medicine* editors asked 7 global health leaders to discuss developments relevant to the equitable provision of medical care to all populations. The result is a collection of expert views on ethical trial design, research during outbreaks, high-burden infectious diseases, diversity in research and protection of migrants.

The *PLOS Medicine* Editors are Clare Garvey, Thomas McBride, Linda Nevin, Larry Peiperl, Amy Ross, Paul Simpson, and Richard Turner.

## Introduction

Despite humanitarian crises and troubling political divides that have threatened the well-being of vulnerable groups throughout 2016, policymakers and researchers continue to pursue the imperative of improving quality and reducing disparities in health. As the year comes to a close, the *PLOS Medicine* Editors have invited expert perspectives on recent progress towards social justice in medicine and priorities for the future. This month’s editorial presents the views of leading thinkers on five such topics: treatment of vulnerable trial participants, dissemination of data and research during outbreaks, strategies for high-burden infectious diseases, promotion of diversity among scientific leaders and research participants, and protection of health in migrant populations.

**Annette Rid is a Senior Lecturer in Bioethics & Society in the Department of Global Health & Social Medicine, King’s College London and a Fellow of the Hastings Center. She currently serves on the Working Group to revise the 2002 International Ethical Guidelines for Biomedical Research Involving Human Subjects of the Council for International Organizations of Medical Sciences (CIOMS)**.

*Which ethical challenges in research on the recent Ebola and Zika epidemics will have the greatest influence on how international health communities study future outbreaks*?

Rigorous research is essential for understanding and addressing epidemics of emerging infectious diseases, but it raises many ethical challenges. One challenge in the recent Ebola and Zika epidemics was how investigators should design clinical trials and, more specifically, how their obligations towards trial participants should influence clinical trial design.

In the Ebola epidemic, there was deep ethical disagreement as to whether investigators were justified in using designs that could enhance a trial’s scientific value but withheld the study intervention from some participants [1]. Ebola had a high fatality rate, and targeted treatments and vaccines for the disease did not exist. Many therefore argued that investigators had an obligation to provide all participants with the study intervention, even if this required making scientific trade-offs—especially in the context of treatment trials [2].

In the Zika epidemic, an ongoing ethical question is whether investigators are justified in using designs that accelerate trial results and enhance their scientific value but expose participants to significant risks [3]. Specifically, deliberately infecting participants with the Zika virus would allow the rapid testing of vaccines while gaining valuable insights into the disease. If the spread of Zika slows, such “disease challenge” trials could also be the only way of testing a vaccine in humans.

Both epidemics raise fundamental ethical questions about how investigators’ obligations towards participants constrain scientific pursuit. How does the obligation to enhance potential clinical benefits place limits on acceptable trial design when participants are in dire need, as many were in the Ebola epidemic? How does the obligation not to expose participants to excessive risks constrain designs to accelerate scientific results, as it might in the Zika epidemic?

Traditional research ethics has some answers to these questions. For example, it is generally assumed that enhancing potential benefits for participants must not undermine the scientific and social value of research [4]. Yet, open questions remain about acceptable scientific trade-offs in situations like the Ebola epidemic. Similarly, it is widely agreed that risks to participants must be minimized and proportionate to any potential clinical benefits for them and/or the social value of the research [4]. Yet, the Zika challenge studies reveal unresolved questions about upper-risk limits in research with competent consenting adults, and the value of research that could justify exposing them to significant risks [5]. The answer to these questions will shape the conduct of clinical research in future disease outbreaks and requires global ethical debate. Importantly, this debate should also encompass investigators’ obligations towards participants in research on public health interventions—which might be more important for safeguarding population health during epidemics than clinical trials.

**Michael A. Johansson is a Research Biologist, Zika Response Modeling Team Lead, and Epidemic Prediction Initiative Founder at the United States Centers for Disease Control and Prevention and a Visiting Scientist at Harvard TH Chan School of Public Health**.

*Which outcomes or consequences from recent epidemics will have the greatest influence on how international health communities prepare for and address future outbreaks*?

The recent, largely unexpected epidemics of Ebola, Zika, Middle East respiratory syndrome (MERS), and chikungunya were all caused by unrelated viruses with different nonhuman hosts, modes of transmission, and clinical presentations. The scale, speed, and diversity of these epidemics highlights the need for an equally scalable, rapid, and agile scientific response to address both short-term needs (e.g., identifying diagnostic tools or assessing disease severity) and longer-term needs (e.g., developing vaccines or other interventions). The scientific community has responded to these epidemics with an unprecedented—and ongoing—shift in the speed and scope of disseminating scientific data and research.

Decades ago scientific analyses of outbreaks were published years later in scientific journals that were generally inaccessible to the public. As a result of these recent outbreaks, scientists, funding agencies, and journals have broadly recognized and embraced rapid, open publication and preprint distribution. Though this transition is still just beginning, it is already creating novel, open scientific discourse in the midst of epidemics, when information is scarce but of particularly high value. Moreover, there has been a clear push to share not just research but data. This revolution is occurring on all levels, from digitizing primary field or clinical data, to sharing genomic sequence or experimental data, to developing standards for compiling and disseminating epidemiological data from local health departments up to the World Health Organization. Improved access to data can directly impact the speed and scale at which we learn about and respond to epidemics.

Open data, open access, and open science support the scientific community directly by facilitating research, engaging new talents, enabling reproducibility, and encouraging others to build upon past work. In the context of an epidemic, where early interventions can potentially save lives, the need to move science and data rapidly on a global scale makes these processes even more important. At its best, epidemic response should be driven by evidence derived from data and science. We are all best served when that process is rapid, open, and leverages the data, minds, and skills of the entire global community.

**Gabriel Leung is Chair Professor of Public Health Medicine and Dean of Medicine at the University of Hong Kong**.

*What 2016 developments from infectious disease research in Asia stand out for their potential to bring the greatest health benefits to the greatest number of people*?

Hepatitis C infects more than 180 million worldwide, with East Asia carrying over 30% of the burden [6]. This year’s Lasker-Debakey Clinical Medical Research Award rightfully gives the nod to the triumvirate who carried out a series of elegant fundamental experiments underpinning the development of anti-hepatitis C therapies. In June 2016, the US Food and Drug Administration approved sofosbuvir/velpatasvir as the first oral, single-tablet, pan-genotypic cure, based on the findings of ASTRAL-1 through -4 that were, in part, carried out in Hong Kong [7]. However, to reach the utilitarian ideal of “the greatest good for the greatest number,” there would need to be a third breakthrough in action research on access to medicines. Overcoming the heterogeneity of country health systems and associated hurdles to ensuring that such cures reach the 55 million East Asian chronic hepatitis C carriers will be as challenging as handling the highly heterogeneous viral genotypes, subtypes, and quasispecies during drug development.

Seasonal influenza continues to cause significant disease burden globally. Although influenza vaccines are updated to keep abreast of this rapidly mutating virus, the virus continues to be unpredictable with newly circulating strains evading strain specificity, leading to vaccine failure. An international team led by Yoshi Kawaoka at the Universities of Tokyo and Wisconsin [8] reported a strategy that may potentially allow us to keep one step ahead of the virus. Using random mutagenesis of the viral hemagglutinin together with human immune sera to mimic in vitro the immune selection pressure that occurs in nature, they were able to anticipate which way the virus was likely to evolve, thereby assisting seasonal vaccine strain selection. Separately, Poon and colleagues examined within-host virus genetic diversity when influenza is transmitted in Hong Kong households and demonstrated that multiple virus strains are cotransmitted in the community [9]. The future antigenic drift variants were found to cocirculate as a minor virus population years before they finally emerge as an epidemic strain. Thus, the vaccine virus candidate of tomorrow is hiding as a minor variant within the influenza strains circulating today; and we now have the means to look for them.

Moving from novel interventions at the frontier of science to retooling tried-and-tested interventions, a joint Hong Kong–Boston team provided evidence to support the World Health Organization’s 5-fold fractional-dose strategy for the Kinshasa vaccination campaign against yellow fever in the summer of 2016 [10]. While there has long been a safe and highly effective live-attenuated vaccine against yellow fever, the global emergency stockpile had already been depleted twice by the Angola outbreak since December 2015. The epidemic has by now spread to neighboring Democratic Republic of Congo and elsewhere. With this new evidence based on modelling, public health authorities can feel confident about implementing dose-sparing strategies that would extend the strained vaccine supply to protect five times more people than otherwise. Jeremy Bentham would surely have approved.

**Hannah Valantine is a Senior Investigator at the United States National Heart, Lung, and Blood Institute and the inaugural National Institutes of Health Chief Officer for Scientific Workforce Diversity**.

*Minority populations are predicted to form a collective majority in the US by 2050. What will it take for diversity in the scientific workforce keep pace*?

Diversity promotes innovation. We know from scientific studies that heterogeneous teams outperform homogeneous ones [11]. Moreover, with diverse, complex health challenges facing us, we need all minds at the table. At the National Institutes of Health (NIH), I am leading efforts to embrace diversity as an opportunity, rather than a problem. I see four diversity challenges that we as a biomedical society need to meet in order to reach our research goals and to improve health for the rich tapestry of people and cultures that is modern America. First, we must look at workforce diversity using a scientific lens—and second, we must insist that all our efforts are informed by data. Which programs and strategies work, and in what contexts? How can successful approaches be shared? Third, we must accept the fact that science is a human endeavor that is affected by sociocultural factors. Often, these influences, like bias, are unintentional. Yet they can make and break careers, and individuals from under-represented groups, including women, are especially vulnerable. Finally, we need to ensure that workforce diversity is sustainable, pointing to the need to engage the private sector and to focus on all environments where scientists make important contributions.

*You trained in cardiology when only two female physicians were practicing this specialty, moved from the British Cardiac Society to the American Heart Association as an early career cardiologist, and recently from academia at Stanford to NIH. For others who experience “newcomer” status in biomedicine, could you share the skills or attitudes that you’ve found most helpful*?

Being “the only one in the room” can be hard. Yet, it’s also an opportunity to accomplish one’s own goals and lift the boat for others. I make a conscientious choice to remain confident, even at the risk of violating social norms that some may consider “overconfident” coming from a woman. Second, be an astute observer and listener. Any environment has its own unique culture, so be ready to adjust, adapt, and tailor the way you communicate. Be aware of stereotypes, as they might pertain to you, but don't let them get in your way. Accomplishing this has a lot more to do with humor and grace than dominance and heavy-handedness! Recognize that it’s critical to invite others to play their part in mentoring, diversity efforts, and community service; these activities are not just the responsibility of women and other under-represented groups. Finally, be who you want to be, and don’t feel like you need to explain why. People from all backgrounds are well-suited not only to careers in clinical, community, and implementation science but also to cutting-edge basic and applied research across all fields including neuroscience, basic-science discovery, and in leading the frontiers of research in medicine and surgery.

**Esteban G. Burchard is a physician–scientist at the University of California, San Francisco (UCSF). He was a member of the Advisory Committee to President Obama’s Precision Medicine Initiative, and is an advisor to the National Academy of Medicine. Sam Oh is the Director of Epidemiology for the UCSF Asthma Collaboratory and the Center for Genes, Environments and Health**.

*In 2016, the NIH made its first funding awards to support enrollment in President Obama’s Precision Medicine Initiative (PMI) Cohort Program. You advised the NIH Director on recruitment of the full range of racial, ethnic, and socioeconomic groups in the United States for this cohort. Do you expect success*?

The PMI Cohort Program set an ambitious goal of recruiting one million volunteers that reflect the diversity of the US. However, to accomplish this goal, the NIH must improve its performance over an earlier, unsuccessful commitment to include minorities in clinical and biomedical research, the 1993 Congressionally mandated NIH Revitalization Act. The poor implementation and lack of enforcement of the NIH Revitalization Act has led to worsened health outcomes among patients and, as a result, greater racial/ethnic health disparities. African American patients, and by proxy their family members, are more likely than Whites to be incorrectly diagnosed as having a serious heart disease, hypertrophic cardiomyopathy, because the diagnostic clinical studies underlying the test for this life-threatening heart condition did not include enough African Americans as healthy controls [12]. Similarly, the widely successful heart drug clopidogrel (Plavix) was developed mostly among Whites yet was successfully marketed in Hawaii, a Pacific Island, despite being ineffective for 45% of Asians and 77% of Pacific Islanders [13]. On the other hand, clinical studies that embrace the ethos of the NIH Revitalization Act ultimately benefit all people. The discovery of *PCSK9* missense mutations in African Americans with unusually low LDL cholesterol allowed for the development of a new class of drugs that have revolutionized cholesterol therapy regardless of race/ethnicity [14].

Racial/ethnic minorities make up more than half of all the children born in the US, and Hispanics/Latinos are one of the largest and fastest growing groups [15]. These children are especially at risk when US funding organizations and scientists are not held accountable for recruiting populations that truly reflect the diversity of America. Asthma is the most common and disparate chronic health condition among children [16]. Asthma prevalence in the US is highest among Puerto Ricans (36.5%), intermediate among Blacks (13.0%) and Whites (12.1%), and lowest among Mexican Americans (7.5%) [17]. Asthma mortality is 5.5-fold higher in Puerto Ricans compared to Mexican Americans [18]. Despite these striking disparities, less than 4.5% of NIH-funded pulmonary studies in the last 20 years have included minority populations [19].

Even more striking is that in the face of our ongoing Genomic Revolution, the percentage of research participants with non-European ancestry in NIH-supported modern genetic studies has only increased from 4% in 2009 to 6% in 2016 [20]. Moreover, a recent Gallup Poll demonstrated that 35% of Americans are seriously concerned about race relations in the US [21]. Consequently, the US cannot afford a PMI that has the potential to worsen racial/ethnic health disparities and potentially exacerbate race relations worldwide.

For the PMI to be successful, inclusion of minorities as both participants and scientists is a must. The people best able to reach minorities are scientists from these communities. Unfortunately, racial and ethnic minorities remain grossly under-represented in clinical and biomedical research, and Asian and minority scientists are less likely to receive NIH research grant support (R01 funding) than White scientists [22–24].

The NIH and clinical and scientific communities need to be held accountable for increasing diversity in research. However, we cannot be expected to fix the problem in the face of declining budgets. Clinical and biomedical research communities need to be given the resources, including adequate budgets, to address these issues. Representing diverse populations in scientific research is important as a matter of social justice, economics, and science. We should embrace diversity such that the rising tide of precision medicine lifts all boats, including groups disproportionately affected by disease and those who have been historically understudied and excluded from opportunities to participate as meaningful scientific leaders and partners in clinical and biomedical research.

**Cathy Zimmerman is a Professor in the Department of Global Health and Development at the London School of Hygiene and Tropical Medicine. Her research focuses on mobile populations, violence, and health**.

*What 2016 developments in health policy-making do you expect will bring the greatest changes in the health care of migrant populations*?

In 2016, we have seen unprecedented attention to vulnerable migrants, especially refugees and victims of human trafficking. With the UN General Assembly Summit on Refugees and Migrants, and the 2030 Sustainable Development Goals that include commitments against labor exploitation, the international community has put the plight of people seeking protection and decent employment squarely on the global agenda [25,26]. Although specific discussions of health are seldom central to these dialogues, reports regularly feature the abuses, deprivation, and fatalities among refugees. For migrant workers, there has been much less discussion of the substantial health and safety risks that workers face in hazardous labor sectors, such as commercial fishing, construction, mining, agriculture, manufacturing, and forced sex work [27].

High-level commitment to migrants breaks important ground for action to protect the health of approximately one billion of the world’s most vulnerable individuals. Unfortunately, good deeds to match these good intentions are potentially hindered by the politics of discrimination and exclusion—most starkly seen amidst recent events in Europe, the UK “Brexit” referendum and the xenophobic rhetoric of the US presidential campaign. Now is a propitious moment for health leaders to show that we all benefit from inclusion, committing their voices, funds, and services to promote people’s good health, regardless of where they come from or where they land.
